# Peripheral adaptive immunity of the triple transgenic mouse model of Alzheimer’s disease

**DOI:** 10.1186/s12974-018-1380-5

**Published:** 2019-01-05

**Authors:** Isabelle St-Amour, Cristina R. Bosoi, Isabelle Paré, Prenitha Mercy Ignatius Arokia Doss, Manu Rangachari, Sébastien S. Hébert, Renée Bazin, Frédéric Calon

**Affiliations:** 1 Axe Neurosciences, Centre de recherche du CHU de Québec-Université Laval, QC, Québec Canada; 2 Département de psychiatrie et neurosciences, Faculté de médecine, Université Laval, QC, Canada; 3 Centre de Recherche de l’IUCPQ-Université Laval, QC, Québec Canada; 4 Medical Affairs and Innovation, Héma-Québec, QC, Québec Canada; 5 Faculté de pharmacie, Université Laval, QC, Québec Canada

**Keywords:** Adaptive immune system, Hematopoietic stem cell, Th17, Inflammation, Alzheimer’s disease, Neurodegenerative disease, Lymphocyte egress

## Abstract

**Background:**

Immunologic abnormalities have been described in peripheral blood and central nervous system of patients suffering from Alzheimer’s disease (AD), yet their role in the pathogenesis still remains poorly defined.

**Aim and methods:**

We used the triple transgenic mouse model (3xTg-AD) to reproduce Aβ (amyloid plaques) and tau (neurofibrillary tangles) neuropathologies. We analyzed important features of the adaptive immune system in serum, primary (bone marrow) as well as secondary (spleen) lymphoid organs of 12-month-old 3xTg-AD mice using flow cytometry and ELISPOT. We further investigated serum cytokines of 9- and 13-month-old 3xTg-AD mice using multiplex ELISA. Results were compared to age-matched non-transgenic controls (NTg).

**Results:**

In the bone marrow of 12-month-old 3xTg-AD mice, we detected decreased proportions of short-term reconstituting hematopoietic stem cells (0.58-fold, *P* = 0.0116), while lymphocyte, granulocyte, and monocyte populations remained unchanged. Our results also point to increased activation of both B and T lymphocytes. Indeed, we report elevated levels of plasma cells in bone marrow (1.3-fold, *P* = 0.0405) along with a 5.4-fold rise in serum IgG concentration (*P* < 0.0001) in 3xTg-AD animals. Furthermore, higher levels of interleukin (IL)-2 were detected in serum of 9- and 13-month-old 3xTg-AD mice (*P* = 0.0018). Along with increased concentrations of IL-17 (*P* = 0.0115) and granulocyte-macrophage colony-stimulating factor (*P* = 0.0085), these data support helper T lymphocyte activation with Th17 polarization.

**Conclusion:**

Collectively, these results suggest that the 3xTg-AD model mimics modifications of the adaptive immunity changes previously observed in human AD patients and underscore the activation of both valuable and harmful pathways of immunity in AD.

## Introduction

Impaired immunity is an undeniable part of Alzheimer’s disease (AD) pathophysiology, although its direct contribution to disease onset and progression is still much debated [1]. The main cellular components of the adaptive immunity are B and T lymphocytes, ultimately responsible for humoral (antigen-specific antibody secretion) and cell-mediated immunity. The adaptive immune response plays a key role in the development of adequate control against pathogens, cancer cells, and toxic molecules including misfolded tau and amyloid-beta peptide (Aβ) proteins [2, 3].

The triple transgenic (3xTg-AD) mouse model displays tau and Aβ (amyloid plaques) accumulation in the brain increasing with age, as well as changes in their immune system [4–8]. In the blood of 3xTg-AD mice, we previously reported that while granulocytes are not significantly affected, total leukocytes, B, and both CD4^+^ and CD8^+^ T lymphocytes are decreased compared with controls [4]. Interestingly, we further observed an increase in CD4/CD8 T lymphocyte ratio suggesting an imbalance between helper/cytotoxic cells [4]. These results suggest an overall deficit in adaptive immune response and are consistent with data reporting aberrant lymphocyte populations in AD individuals [9–14].

To better decipher the causes and consequences of these modulations on immune response, we investigated hematopoietic cells in primary (bone marrow) and secondary (spleen) lymphoid organs along with in vivo lymphocyte activation cues from blood cytokine and immunoglobulin G (IgG) production in the 3xTg-AD model. We observed decreased levels of short-term reconstituting (STR) hematopoietic stem cells in the bone marrow. Furthermore, results from immunoglobulin G (IgG) and cytokine quantification suggest increased B and T lymphocyte activation respectively. Finally, elevated levels of interleukin (IL)-2, IL-17 and granulocyte-macrophage colony-stimulating factor (GM-CSF) strongly point to Th17 polarization in the 3xTg-AD model of tau and Aβ neuropathologies.

## Material and methods

### Animals, treatment and tissue preparation

The 3xTg-AD mouse model of AD used in this study was developed by Oddo and colleagues [15] and bred in our animal facility. These mice harbor three mutant genes, namely genes coding for the human beta-amyloid precursor protein (APP), tau (in Thy1.2 expression cassettes), and presenilin-1 (PS1, knockin) with human mutations from familial AD (APP_swe_, PS1_M146V_) and frontotemporal dementia (tau_P301L_). The 3xTg-AD replicates many features of AD including Aβ and tau pathologies as well as cognitive deficits. Previous studies showed that 3xTg-AD mice fully develop neuropathological and behavior changes around 12 months of age [6, 7]. The non-transgenic (NTg) controls used in this study were generated from the backcross of our 3xTg-AD colony with B6126SF1/J animals, maintained on a mixed B6129 background and bred in our animal facility. The Laval University Animal Research Committee (Québec, QC, Canada) approved all procedures. The animals used for post-mortem analyses of adaptive immunity are the control group (vehicle) from preclinical evaluation of human intravenous immunoglobulin (IVIg) efficacy reported earlier and included 8 NTg and 12 3xTg-AD animals, 50% females in each group [4]. These animals received intraperitoneal administrations of sterile 0.2 M glycine pH 4.25, endotoxin free, twice a week for 3 months (27 injections). Mice were killed when overt AD-like neuropathologic changes are observed (12 ± 0.1 months) under deep anesthesia (100 mg/kg ketamine, 10 mg/kg xylazine) via terminal intracardiac perfusion of PBS containing protease and phosphatase inhibitors. Serum was prepared from intracardiac blood, and splenocytes and bone marrow (both tibia and femur) cells were isolated and stored in liquid nitrogen until used. Cytokines and chemokines were quantified before and after the development of overt AD-like neuropathological processes: blood from untreated 9- (9.0 months ± 0.1) and 13- (12.9 months ± 0.2) month-old mice (*N* = 7–8 mice per group, 4 females in each group) was drawn from the saphenous vein, clotted on ice, and centrifuged at 5000 g for 5 min for serum recovery. Serum was frozen at − 80 °C until used.

### Reagents

Unless otherwise specified, all biochemical reagents were purchased from JT Baker (Phillipsburg, NJ, USA).

### Flow cytometry analysis

To study cell marker expression, the following fluorochrome-conjugated antibodies were used as described [4, 16]: anti-CD45 (leukocyte marker; clone 30-F11; eBioscience, San Diego, CA, USA), anti-CD3 (T lymphocyte marker; clone 17A2; BD Biosciences, Mississauga, ON, Canada), anti-B220 (B lymphocyte marker; clone RM4-5; BD Biosciences), anti-CD4 (helper T lymphocyte marker; clone GK1.5; eBioscience), anti-CD8b (cytotoxic T lymphocyte marker; clone eBioH35-17.2; eBioscience), anti-CD25 (regulatory T lymphocyte marker; clone PC61.5; eBioscience), anti-Foxp3 (regulatory T lymphocyte marker; clone FJK-16 s; eBioscience), anti-F4/80 (macrophage marker; clone BM8; eBioscience), anti-CD11b (macrophage and granulocyte marker; clone M1/70; eBioscience), anti-Gr1 (granulocyte marker; Ly-6G, Clone RB6-8C5; eBioscience), or relevant isotypic controls in PBS-1%BSA. We used 7-amino-actinomycin D to assess cell viability (eBioscience). Cells were acquired and analyzed using a CyFlow ML cytometer (Partec North America, Inc., Swedesboro, NJ, USA) and FCS Express software (De Novo Software, Los Angeles, CA, USA).

Hematopoietic stem cells were quantified in bone marrow with the Mouse Hematopoietic Stem Cell Isolation Kit (BD Biosciences). It includes a lineage cocktail (lin), which contains antibodies against CD3, CD11b, B220, Ly6C, Gr1, and TER-119 to exclude differentiated cells. Both long-term reconstituting (LTR) and STR hematopoietic stem cells express the cell markers Sca-1 and c-Kit, whereas only STR expresses CD34. Therefore, lin^−^/Sca-1^+^/cKit^+^/CD34^−^ cells are LTR and lin^−^/Sca-1^+^/cKit^+^/CD34^+^ cells are designated STR.

### Enzyme-linked immunosorbent assay quantification

A mouse IgG-specific enzyme-linked immunosorbent assay (ELISA) was performed to assess IgG concentration in serum and cortex using goat anti-mouse IgG Fc-specific antibodies (Jackson ImmunoResearch Laboratories Inc., West Grove, USA) [17]. Cytokine and chemokine concentrations were determined using a multiplex ELISA (Q-plex Mouse Cytokine—Screen (16-plex); Quansys Bioscience, Logan, UT, USA). The following cytokines and chemokines were analyzed: IL-1α, IL-1β, IL-2, IL-3, IL-4, IL-5, IL-6, IL-10, IL-12p70, IL-17, tumor necrosis factor (TNF)α, granulocyte-macrophage colony-stimulating factor (GM-CSF), regulated on activated, normal T cell expressed and secreted (RANTES), monocyte chemoattractant protein-1 (MCP-1, also called CCL2), and macrophage inflammatory protein 1α (MIP-1α).

### Enzyme-linked immunosorbent spot quantification

Splenocytes isolated using dissociation were thawed, washed, counted, and plated on mouse anti-IgG-coated wells (Multiscreen®HTS filter plate; Millipore Corporation, Billerica, MA, USA) and left immobile for antibody secretion overnight at 37 °C. After washing the cells, wells were incubated with horseradish peroxidase-conjugated goat anti-mouse IgG Fc-specific antibody and mouse immunoglobulins were detected with TrueBlue peroxidase substrate (VWR, Ville Mont-Royal, QC, Canada). Each spot was counted under a dissection microscope and considered as a single IgG-secreting B cell.

### Immunofluorescence

At sacrifice, a separate set of 12-month-old mice (*N* = 3 NTg and 6 3xTg-AD, treated with glycine as described above) were perfused with PBS, brain hemispheres were recovered, fixed in 4% paraformaldehyde for 48 h then cryoprotected in 20% sucrose at 4 °C for > 72 h. Then, 25-μm-thick sections were cut and stained as previously described [4, 17]. For detection of cerebral IgG and amyloid plaques, free-floating sections were blocked with 5% normal horse serum, 0.2% Triton X-100, and 2.4G2 antibody (to block mouse Fc receptors) in PBS. Sections were incubated overnight at 4 °C, with an Alexa-Fluor 568 conjugated goat anti-mouse IgG antibody. After the overnight incubation, sections were washed in PBS and incubated for 2 h with an Alexa-Fluor 568 donkey anti-goat antibody (Life Technologies, Burlington, ON, Canada) at room temperature. The sections were further washed and placed in a 4′,6-diamino-2-phenylindole (DAPI) for 7 min to stain the nuclei, then mounted on slides, incubated for 5 min with a 0.1% thioflavin-S solution to stain amyloid plaques, and treated with 0.5% Sudan Black to minimize the autofluorescence. After drying, the slides were coverslipped in Fluoromount mounting media. Images were acquired using a Zeiss AxioImager M2 microscope. Analysis was performed with ZEN 2012 SP2 and ImageJ 1.51 s Software in the hippocampus [4].

### Statistical analyses

Statistical analyses were performed using Prism 7.0a (GraphPad Software Inc., San Diego, CA, USA) and JMP 14 (SAS, Marlow, Buckinghamshire, UK). The threshold for statistical significance was set to *P* < 0.05. Homogeneity of variance and normality was determined for all data sets using D’Agostino & Pearson’s normality test. When normality was verified, unpaired *t* test was performed. When needed, logarithmic transformation was performed to reduce variances and provide more normally distributed data. Otherwise, Mann-Whitney was applied when Gaussian distribution was not confirmed. For cytokine quantification, we consulted the Statistical Consulting Service from the Université Laval. To compare the effect of the transgenes in two age groups, we used two-way ANOVA when normality of the residuals was confirmed. When normality was not confirmed, data were analyzed first using Kruskal-Wallis test followed by Dunn’s multiple comparison. Then to identify the global effect of genotype, results from the two ages were grouped and analyzed using Mann-Whitney test. All statistical analyses are described in Table 1.

**Table 1:** Description of statistical analyses

Figure referenced	Statistical analysis	*P* value	Differences between means or medians^a^
1A	Unpaired *t* test	*P* > 0.05	
1B (except short-term reconstituting stem cells)	Unpaired *t* test	*P* > 0.05	
1B—short-term reconstituting stem cells	Mann-Whitney test	*P* = *0*.*041*	Median A = 0.155; Median B = 0.075; Difference (Hodges-Lehmann): − 0.06
3A—serum IgG	Mann-Whitney test	*P* < *0*.*0001*	Median A = 0.77; Median B = 4.505; Difference (Hodges-Lehmann): 3.33
3A—cortical IgG	Mann-Whitney test	*P* = *0*.*0002*	Median A = 0.4287; Median B = 0.9211; Difference (Hodges-Lehmann): 0.5244
3A—IgG-secreting B lymphocytes	Unpaired *t* test	*P* = 0.059	
3A—Plasma cells	Mann-Whitney test	*P* = *0*.*041*	Median A = 1.09; Median B = 1.505; Difference (Hodges-Lehmann): 0.385
4—IL-1β, IL-3, MCP-1, RANTES	Two-way ANOVA	*P* > 0.05	
4—IL-1α, IL-4, IL-5, IL-6, IL-10, IL-12p70, MIP-1α	Kruskal-Wallis test Mann-Whitney test (NTg vs. 3xTg-AD)	*P* > 0.05 *P* > 0.05	
4—IL-2	Kruskal-Wallis test		Not applicable
	Dunn’s multiple comparison:	*P* = *0*.*0256*	
	9 months NTg vs. 3xTg-AD	*P* = 0.0816.	
	13 months NTg vs. 3xTg-AD	*P* = 0.0530	
4—IL-2	Mann-Whitney test (NTg vs. 3xTg-AD)	*P* = *0*.*0018*	Median A = 7.5; Median B = 108.7; Difference (Hodges-Lehmann): 84.9
4—IL-17	Two-way ANOVA		Not applicable
	Interaction	*P* = 0.8262	
	Age	*P* = 0.7464	
	Genotype	*P* = *0*.*0115*	
4—GM-CSF	Kruskal-Wallis test		Not applicable
	Dunn’s multiple comparison:	*P* = *0*.*0460*	
	9 months NTg vs. 3xTg-AD	*P* = *0*.*0246*	
	13 months NTg vs. 3xTg-AD	*P* = 0.4916	
4—GM-CSF	Mann-Whitney test (NTg vs. 3xTg-AD)	*P* = *0*.*0085*	Median A = 88.4; Median B = 524; Difference (Hodges-Lehmann): 347
4—TNFα	Kruskal-Wallis test		Not applicable
	Dunn’s multiple comparison:	*P* = *0*.*0135*	
	9 months NTg vs. 3xTg-AD	*P* = *0*.*0179*	
	13 months NTg vs. 3xTg-AD	*P* = 0.3412	
4—TNFα	Mann-Whitney test (NTg vs. 3xTg-AD)	*P* = *0*.*0050*	Median A = 18.4; Median B = 86.9; Difference (Hodges-Lehmann): 65.2

*GM-CSF* granulocyte-macrophage colony-stimulating factor, *IL* Interleukin, *MCP-1* monocyte chemoattractant protein-1, *NTg* non-transgenic animals, *RANTES* regulated on activated, normal T cell expressed and secreted; TNF-α, tumor necrosis factor α

Parametric tests were used only when normality was verified using D’Agostino & Pearson’s normality test. For cytokine/chemokine analysis (Fig. 4), two-way ANOVA was performed when normality of the residuals was confirmed (IL-1β, IL-3, IL-17, MCP-1, and RANTES). When normality was not confirmed, cytokine/chemokine data were analyzed first using Kruskal-Wallis test followed by Dunn’s multiple comparison. Then to identify the global effect of genotype, results from the two ages were grouped (*N* = 15 NTg; *N* = 16 3xTg-AD) and analyzed using Mann-Whitney test. Significant *P*-values are italicized

^a^For comparison between groups; significant data only.

## Results

### Reduction of blood lymphocyte numbers in 3xTg-AD mice could result from multipotent progenitor decline in the bone marrow

**Fig. 1:**
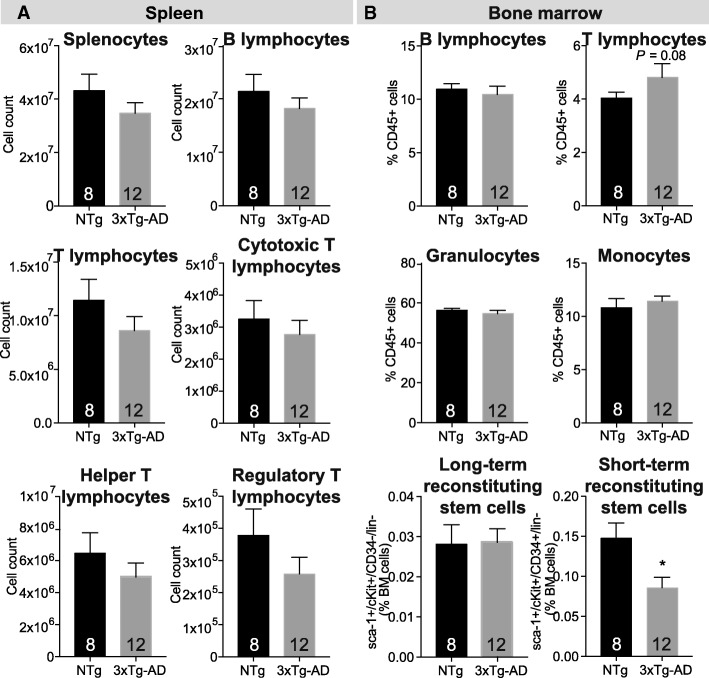
Leukocyte populations in lymphoid organs of the triple transgenic (3xTg-AD) model. **a** Quantification of leukocyte and lymphocyte subpopulations in the spleen of 3xTg-AD mice by flow cytometry revealed similar concentrations of B and T lymphocytes compared to non-transgenic animals (NTg). **b** Enumeration of bone marrow cells indicates comparable proportions of B and T lymphocytes, monocytes, granulocytes, and long-term reconstituting (LTR) but reduction of short-term reconstituting (STR) hematopoietic stem cells. Data are presented as mean ± SEM (*N* = 8 NTg and *N* = 12 3xTg-AD 12-month-old animals per group, *N* are indicated on the graphs). Statistical analysis: Refer to Table 1. Mann-Whitney test (non-parametric) and Welch’s *t* test (parametric) was performed. *P = 0.0116 compared to NTg

**Fig. 2:**
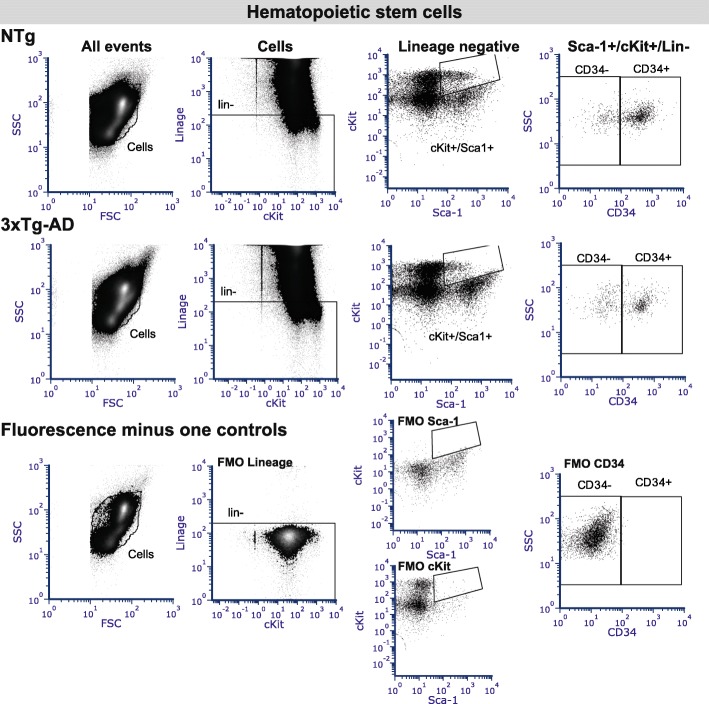
Representative flow cytometry plots for bone marrow hematopoietic stem cells. Long-term reconstituting (LTR, lin^−^/Sca-1^+^/cKit^+^/CD34^−^) and short-term reconstituting (STR, lin^−^/Sca-1^+^/cKit^+^/CD34^+^) hematopoietic stem cells were quantified in bone marrow. The debris and dead cells were excluded before the selection of lineage negative cells. The Sca-1^+^/cKit^+^ double-positive cells were then gated. Finally, LTR and STR were separated according to their expression of the CD34 marker. Representative results from (*upper panels*) non-transgenic (NTg) and (*center panels*) 3xTg-AD mice are presented. (*Lower panels*) The fluorescence minus one (FMO) controls were used for appropriate gating of the cell populations

To determine if the decrease in lymphocytes previously observed in 3xTg-AD blood [4] was generalized to primary and secondary lymphoid organs, we evaluated the cell populations of hematopoietic cells extracted from bone marrow and spleen. Comparable numbers of T and B lymphocytes as well as T subpopulations (helper, cytotoxic, and regulatory T lymphocyte) were detected in the spleen of 3xTg-AD mice compared to age-matched NTg controls (Fig. 1a). Similarly, in the bone marrow of 3xTg-AD animals, the levels of lymphocytes but also monocytes and granulocytes were unchanged (Fig. 1b). In humans, reduction in hematopoietic stem cell number is observed in early AD and correlates negatively with age and cerebrospinal fluid Aβ42/40 ratio [18]. To determine whether hematopoietic stem cell population could explain the decreased number of lymphocytes observed in the blood of 3xTg-AD mice, we analyzed the hematopoietic stem cell progenitor populations in the bone marrow using Sca-1, c-kit, and CD34 expression on Lin− cells. Lin− cells do not express markers normally present on lineage committed hematopoietic cells. We quantified LTR and STR hematopoietic stem cells using Sca-1^+^/cKit^+^/CD34^−^/lin^−^ and Sca-1^+^/cKit^+^/CD34^+^/lin^−^ cells, respectively [19, 20] (Fig. 1b and Fig. 2). Although the proportions of LTR hematopoietic stem cells were similar between groups, we observed a significant decrease of STR in 3xTg-AD animals (0.58 fold, *P* = 0.0116, Fig. 1b). These cells are precursors of common myeloid progenitors and common lymphoid progenitors, which migrate to secondary lymphoid organs and produce lymphoid differentiated cells [21]. The diminution in STR observed here, likely the lymphoid-committed progenitors, could explain decreasing levels of circulating B and T lymphocytes observed in the blood of 3xTg-AD mice.

### Increased B lymphocyte activation in vivo in 3xTg-AD mice

**Fig. 3:**
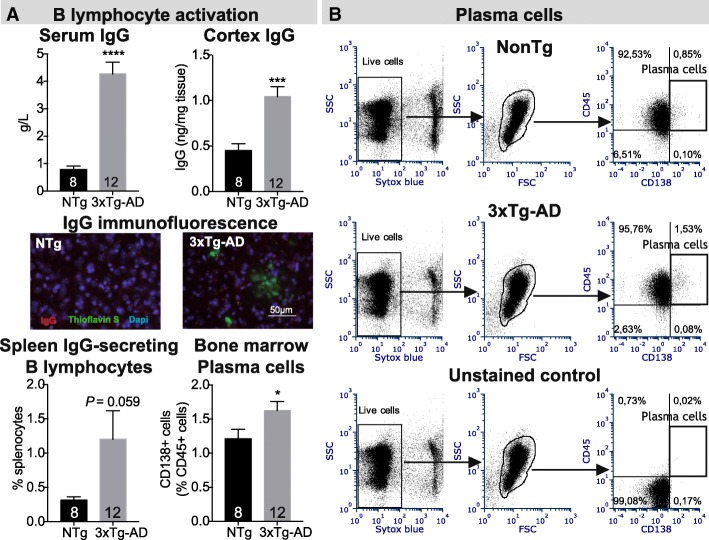
Increased activation of B lymphocytes in the 3xTg-AD mouse. **a (***Upper panels*) The rise of serum and cortex immunoglobulin G (IgG) concentrations (ELISA), and (*lower panels*) percentage of IgG-secreting cells (ELISPOT, spleen) and plasma cells (flow cytometry, bone marrow) strongly suggest elevated activation of B lymphocyte in the 3xTg-AD model compared to non-transgenic animals (NTg, *N* = 8–12 12-month-old mice per group. *N* are indicated on the graphs). (*Center panels*) Representative immunofluorescence staining of IgG (*red*), amyloid plaques (thioflavin-S, *green*) and nuclei (DAPI, *blue*) from the hippocampus of NTg and 3xTg-AD mice are presented. Statistical analysis: Refer to Table 1. Mann-Whitney test, *P < 0.05; ***P < 0.001; ****P < 0.0001 compared to NTg. **b** Representative flow cytometry plots for CD138+ bone marrow plasma cells populations

Activation and maturation of immune cells are tightly regulated to ensure adequate immune response while protecting from autoimmune response. To evaluate lymphocyte activation, we first determined the serum IgG concentration. Interestingly, we observed a 5.4-fold increase in IgG concentration in 3xTg-AD mice (*P* < 0.0001, Fig. 3a). We also detected a 2.3-fold rise of IgG levels in the cortex of 3xTg-AD mice (*P* = 0.0002) but no co-localization with amyloid plaques was observed (Fig. 3a). To confirm the increase B lymphocyte activation, we next assessed the proportion of IgG-secreting B lymphocytes among total splenocytes using enzyme-linked immunosorbent spot (ELISPOT) quantification. We detected a trend toward an increase in IgG-secreting cells in the spleen (3.8-fold, *P* = 0.059, Fig. 3a). Because mature B lymphocytes (plasma cells) are responsible for the production of large amounts of immunoglobulins, we next measured the number of plasma cells (CD138^+^ lymphocytes) in bone marrow and observed a 1.3-fold increase of CD138^+^ cells in 3xTg-AD mice compared to controls (*P* = 0.0405, Fig. 3). Because sex differences in behavior and Aβ load have been observed in the 3xTg-AD model [6, 22–24], we further analyzed males and females separately (data not shown). Albeit low statistical power, we did not observe major sex differences. Indeed, although a trend toward more numerous plasma CD138+ cells was observed in female mice only, higher IgG levels were noted in the serum and cortex of both males and females, suggesting that the overall activation of B lymphocytes is not sex dependent. Taken together, these results provide evidence of B lymphocytes activation in the 3xTg-AD mouse model.

### Cytokine profiling supports increased activation along with Th17 polarization of T lymphocytes

**Fig. 4:**
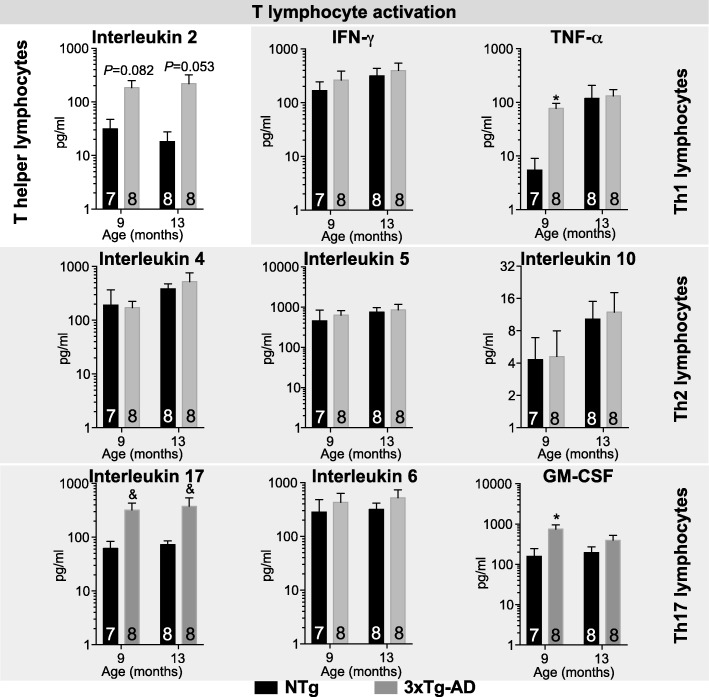
Increased activation of T lymphocytes in the 3xTg-AD mice. Levels of serum cytokines/chemokines were determined by multiplex ELISA (*N* = 7–8 9- and 13-month-old animals per group. *N* are indicated on the graphs). Molecules detected include interleukin (IL)-1α, IL-1β, IL-2, IL-3, IL-4, IL-5, IL-6, IL-10, IL-12p70, IL-17, tumor necrosis factor α (TNFα), granulocyte-macrophage colony-stimulating factor (GM-CSF), regulated on activated, normal T cell expressed and secreted (RANTES), monocyte chemoattractant protein-1 (MCP-1), and macrophage inflammatory protein 1α (MIP-1α). Increased levels of IL-2 were observed in 3xTg-AD mice and characterize T lymphocyte activation. Cytokines secreted by T helper lymphocytes (Th), Th1, Th2, and Th17 are presented and support Th17 polarization. All the other cytokines/chemokines listed above were unchanged between groups. Data are presented as mean ± SEM. Statistics: &, *P* < 0.05 Effect of genotype determined by two-way ANOVA; * *P* < 0.05 Kruskal-Wallis test followed by Dunn’s multiple comparison test NTg vs. 3xTg-AD at each different age; refer to Table 1 for more details

Serum cytokine quantification using multiplex ELISA probing in 9- and 13-month-old 3xTg-AD animals revealed increased levels of IL-2, TNF-α, IL-17, and GM-CSF compared with controls but no changes in other cytokine/chemokine investigated (Fig. 4 and data not shown, detailed statistics: Table 1). Interestingly, from the panel of cytokine/chemokine tested, IL-2 is secreted almost exclusively by activated CD4^+^ T helper lymphocyte [25]. Moreover, IFN-γ, TNF-α, and IL-2 are secreted by Th1 T lymphocyte; IL-4, IL-5, and IL-10 are the signature of Th2 lymphocytes, whereas IL-2, IL-6, IL-17, and GM-CSF relates to Th17 activation [26–28]. Therefore, our results suggest increased activation of T helper lymphocytes along with Th17 polarization in the 3xTg-AD model, particularly in 9-month-old mice.

## Discussion

In this study, we sought to deepen our understanding of the relationship between adaptive immune-related impairments and AD neuropathology, using the 3xTg-AD mouse model. Lymphocyte proportions were not changed in the primary and secondary lymphoid organs investigated, but the concentrations of hematopoietic STR in the bone marrow were decreased. We also reported evidence of increased B and T lymphocyte activation along with Th17 polarization in the 3xTg-AD mice, before the overt accumulation of Aβ and tau pathologies. These changes also occurred in the absence of Aβ and tau genetic expression in immune cells, consistent with a crosstalk between the CNS and peripheral immune cells. Interestingly, some of these modifications have also been described in AD patients (Table 2), validating this animal model for the study in immune changes in AD.

**Table 2:** Comparison of adaptive immunity defects observed in the 3xTg-AD model and human AD

Immunologic modification	3xTg-AD mice	AD patients
Hematopoietic stem cells	Decreased bone marrow multipotent progenitors^a^	Reduced circulating CD34^+^ hematopoietic stem cells [18]
B lymphocytes	Increased plasma cells in bone marrow^a^ Decreased in circulation [4]	Decreased in circulation [11, 72, 73]
B lymphocyte antibody secretion	Increased IgG^a^	Increased IgG [74]
T lymphocytes	Decreased circulating helper (CD4) and cytotoxic (CD8) lymphocytes [4] Increased Th17 polarization^a^	Decreased [9, 14, 72, 73, 75]; no change [11, 74] Increased circulating Th17 lymphocytes in early AD [47]
CD4^+^/CD8^+^ ratio	Increased [4]	Increased [14, 76]; no change [72, 73, 75]; decreased [9]
Plasma IL-1α concentration	Decreased [59]	Decreased [77, 78]; no change [75]
Other plasma cytokines	Increased IL-2, IL-17 and GM-CSF^a^ Increased IL-12, decreased IL-1β, IL-5, IL-6, IL-17, TNF-α, IFN-γ, CCL2, CCL3, CCL5, CCL11, and GM-CSF [59]	Increased GM-CSF [76, 79] Increased or no change in IL-1, IL-6, IL-10; Increased IL-4, Il-12, IL-16, Il-18; decreased, increased, or no change in TNF-α (for review see [80])

*3xTg-AD* triple-transgenic mouse model of Alzheimer’s disease, *AD* Alzheimer’s disease, *IgG* immunoglobulin *G; GM-CSF* granulocyte-macrophage colony-stimulating factor, *IL* interleukin

^a^Current paper

Cell surface markers of hematopoietic progenitors are different between humans and mice. In humans, cells expressing the cell surface antigen CD34 are capable of reconstituting long-term, multi-lineage hematopoiesis [29, 30]. Numbers of CD34^+^CD45RO^low^ hematopoietic stem cells were found to be lower in the blood of 23 individuals with early AD compared to 25 Controls [18]. Interestingly, reduced common lymphocyte progenitors are also observed in aged normal mice [31, 32]. Therefore, decreased levels of STR reported here could reflect premature aging of the immune system in the 3xTg-AD model, and suggest that Aβ/tau pathological changes progressively developing in the brain can have an impact on immunological readouts in the periphery.

Antigen presentation, maturation of immunocompetent lymphocytes, and expansion of specific T and B lymphocytes take place in secondary organs, with the lymph nodes funneling lymph and the spleen filtering blood-derived antigens [33]. In AD, Aβ peptides and tau protein have been detected in blood and/or lymph where they can migrate to secondary lymphoid organs and trigger lymphocyte activation [34–39]. Recent research suggests that the meningeal lymphatic system and the cervical lymph nodes play a key role in the clearance of cerebral Aβ peptide [36, 40]. Increased naïve and decreased effector T cells (both CD4^+^ and CD8^+^) were reported in the deep cervical lymph nodes of 5xFAD mice along with increased CD8^+^ effector cells in their brains [41]. Animal models of cerebral amyloidosis present T cell infiltration in the brain, which does not associate with beta-amyloid plaques [42]. In contrast however, T cells have not been detected in the brains of 3xTg-AD mice [43]. In a previous study, we reported a decrease of T lymphocytes in the blood of 3xTg-AD mice [4], associated with higher GM-CSF, IL-12, and IL-5 brain concentrations. Although IL-5 and GM-CSF can be secreted by T lymphocytes, levels of more T-specific cytokines such as IL-2 or IL-17 remained similar to NTg [4]. Therefore, more extensive studies are needed to clarify the role of cerebral T cells in AD pathology.

The increased activation of lymphocytes observed in 3xTg-AD mice could reflect engagement of the adaptive immune response to the removal of AD-related toxic proteins [2]. In agreement with this, we observed higher IgG concentrations in the cortex of 3xTg-AD mice, although no accumulation was seen in amyloid plaques. However, chronic antigenic stress can lead to immune exhaustion [10]. Therefore, immunotherapies against Aβ and tau proteins could gain from the use of both active and passive immunization strategies in order to maintain the immune balance [44–46]. In line with our results, increased activation of circulating lymphocytes [10, 11] together with lower number of naive T lymphocytes [9, 10, 12] were reported in AD patients. Interestingly, cytokine quantification suggests Th17 polarization following helper T lymphocyte activation. Increased circulating Th17 lymphocytes have been reported in early AD [48]. These cells are associated with immunopathogenesis of autoimmune disorders and could promote neuroinflammation in AD [12, 41].

The 3xTg-AD mouse was generated from presenilin 1 (PS1_M146V_) knockin embryos co-microinjected with APP_swe_ and tau_P301L_ Thy1.2 constructs [7, 15]. While transgene expression of tau and Aβ is limited to the brain and spinal cord [15], Aβ in the blood or peripheral organs has also been detected in this model [6, 34, 36, 49]. Increased circulating tau is also detected in AD patients [50–52]. It can thus be speculated that transport of Aβ and tau to the periphery induced an abnormal adaptive immunity response. Moreover, immune cells can cross the blood-brain barrier in AD and induce a cerebral immune response, which may lead to or sustain peripheral immune changes [53]. In any cases, these observations in the 3xTg-AD model lend support to the hypothesis that AD neuropathology may play a causal role in anomalies of peripheral adaptive immunity.

On the other hand, it should be noted that the human presenilin 1 protein expressed in the 3xTg-AD model is under the control of its murine endogenous promoter. Interestingly, in the immune system, presenilins have been implicated in proliferation and signal transduction events in B lymphocytes as well as in thymocytes apoptosis, T lymphocyte expansion, and cytokine production [54–56]. A recent study further demonstrated that, following oxidative stress, the lymphocytes isolated from individuals with familial AD-associated presenilin 1 mutations showed lower depolarization of mitochondrial membrane along with decreased apoptosis rate compared to lymphocytes from sporadic AD [57]. In addition to potential tau/Aβ-related immune activation, the expression of mutant presenilin 1 in immune cells could therefore trigger some of the lymphocyte impairments observed here.

Previous results from spleen lymphocyte quantification in this model have yielded controversial data. For example, reduced [8], unchanged [58], and increased [59] levels of T lymphocytes have been reported in the spleen of 2- and 12-month-old males, 4-month-old males, and 14- and 24-month old 3xTg-AD mice (males and females), respectively. In the study by Yang and colleagues, which reported increased levels of T lymphocytes, the investigators used C57Bl/6 controls instead of B6129 mice [59]. The age, sex, and exact controls used in each study could therefore explain some differences observed.

In contrast with our previous results based on blood analyses [4], we did not observe lower levels of lymphocytes in the primary and secondary lymphoid organs investigated in 12-month-old 3xTg-AD mice. In healthy humans, lymphocytes present in the blood only account for approximately 2% of the total lymphocyte pool; the other 98% being distributed throughout the body [60]. Their mean transit time in the blood is evaluated to about 30 min compared to several hours in secondary lymphoid organs such as the spleen [60, 61]. Therefore, small, statistically undetected alterations in lymphocyte composition in the spleen and bone marrow could cause major alterations in the blood [60].

Discrepancy between lymphocyte concentrations in blood and lymphoid organs between 3xTg-AD and NTg mice could also be explained by deficient egress in the 3xTg-AD animals. Sphingosine-1-phosphate (S1P), a lipid mediator, has been identified as the driving force that mediates egress of lymphocytes from lymphoid organs depending on S1P concentration gradient, which is low in lymphoid organs and high in blood and lymph [33, 61, 62]. In AD, levels of S1P are reduced in human brain samples [63, 64], whereas it has been shown to protect cultured cortical neurons against Aβ toxicity [65]. S1P receptors S1P_1_, S1P_2_, and S1P_3_ are expressed in cerebral endothelial cells [66] and regulate barrier integrity, which is critical to the control of central nervous system inflammation (reviewed in [67]). Therefore, impaired S1P signaling could exacerbate the neuropathological progression in AD models. FTY720 is an agonist of S1P receptor (S1PR). Although it causes the depletion of circulating lymphocytes, treating 5xFAD mice with FTY720 decreases levels of Aβ peptides in the frontal cortex along with reduction of activated microglia [68]. However, the lower dose (1 mg/kg/day) was more effective than the higher dose (5 mg/kg/day), suggesting that suboptimal S1PR agonist could be preferred in AD therapy [68]. In AD, egress impairments could also result from increased cortisol. Indeed, whereas plasma cortisol concentrations are higher in AD [69, 70], a robust rise of plasma cortisol in a mouse model of acute traumatic brain injury was linked to transient lymphocytopenia that was reversed by injection of S1P or rolipram, highlighting a complex and tightly regulated mechanism of lymphocyte egress [71].

## Conclusion

In conclusion, our data show that a significant proportion of adaptive immunity defects observed in human AD are recapitulated in the 3xTg-AD model (Table 2), suggesting a causal role of typical Aβ and tau pathologies. These alterations include modifications interpreted as both beneficial and detrimental, highlighting the complex and delicate balance between adequate antibody-directed removal of pathologic proteins and adverse autoimmune response of the adaptive immune system in AD.

## Abbreviations

3xTg-AD: Triple transgenic mouse model of Alzheimer’s disease
AD: Alzheimer’s disease
APP: Beta-amyloid precursor protein
Aβ: Amyloid-beta peptide
ELISA: Enzyme-linked immunosorbent assay
ELISPOT: Enzyme-linked immunosorbent spot
GM-CSF: Granulocyte-macrophage colony-stimulating factor
IgG: Immunoglobulin G
IL: Interleukin
IVIg: Intravenous immunoglobulin
lin: Lineage cocktail
LTR: Long-term reconstituting hematopoietic stem cells
MCP-1 (CCL2): Monocyte chemoattractant protein-1
MIP-1α: Macrophage inflammatory protein 1α
NTg: Non-transgenic controls
PS1: Presenilin-1
RANTES: Regulated on activated, normal T cell expressed and secreted
S1P: Sphingosine-1-phosphate
S1PR: Sphingosine-1-phosphate receptor
STR: Short-term reconstituting hematopoietic stem cells
TNFα: Tumor necrosis factor alpha
